# Exploring Changes in Barriers and Facilitators for Physical Activity during the Retirement Transition: A Qualitative Interview Study Based on the Behavior Change Wheel

**DOI:** 10.1155/2024/3257287

**Published:** 2024-09-16

**Authors:** Nina Vansweevelt, Jannique van Uffelen, Filip Boen, Jan Seghers

**Affiliations:** Department of Movement Sciences KU Leuven, Leuven, Belgium

## Abstract

The retirement transition has been associated with a decrease in total physical activity (PA). However, little is known about effective interventions to counteract this decrease. Prior to designing interventions, more information is needed about the changes in PA and in determinants of PA around this life change. Therefore, the first aim of this study was to investigate perceived changes in PA. The second aim was to explore the changes in PA barriers and facilitators experienced by recent retirees. Semistructured interviews were conducted with 30 retirees six to ten months after their retirement transition. The interview guide consisted of open questions as well as specific questions based on the “behavior change wheel” (BCW). The analysis of the interviews involved an initial inductive reflexive thematic analysis, followed by deductive mapping of the themes onto the COM-B categories, which are an integral part of the BCW. Most retirees experienced an increase in PA. The first inductive theme regarding changes in barriers and facilitators for PA was labelled as “changes in time availability and time structure.” For instance, one of the facilitators was that increased time availability led to more PA. However, as a barrier, it was noted that this sometimes resulted in increased procrastination as well. The second theme was labelled “emotional/mental changes” and included facilitators such as a decreased feeling of being useful with consequent uptake of new purposeful (physical) activities. Conversely, a perceived barrier was the idea that reduced PA is allowed after retirement. The third theme, “social changes,” highlights for instance the facilitator that many participants expressed an increased desire to expand their social network for shared (physical) activities. The identified themes were mapped onto the COM-B categories and potential intervention functions were discussed.

## 1. Introduction

The world population is aging rapidly and this poses major challenges to maintaining social and health systems [1]. Therefore, it is important to ensure healthy aging by improving PA levels [2–4]. The transition to retirement could be an interesting opportunity to start or to maintain a physically active lifestyle. However, several studies found that in general, during the retirement transition, total PA decreases [5–7], whereas time spent in specific domains of PA, such as recreational PA and domestic/household PA, increases [5, 8]. These changes seem to differ between socioeconomic groups, with higher socioeconomic groups having more favourable changes in PA during the retirement transition, in particular regarding total PA, occupational PA, recreational PA, and active transport [9]. Taking into account the aging society and the beneficial health effects of PA [2–4], it is important to understand the impact of retirement on PA and its determinants.

Previous qualitative studies identified several PA facilitators that were perceived to change during the retirement transition: an increase in time availability [10]; increased energy levels [10]; more flexibility to plan PA, for example during the daytime [10]; an increased need for social interaction [11]; the need for a new personal challenge and/or a new routine [11]; and an increased awareness of the importance of PA for one's own aging [11]. Barriers that were perceived to change during the retirement transition were increased procrastination [10], loss of daily structure [10, 11], a decrease in financial resources and an increased awareness of the cost of PA [10], increased social responsibilities such as voluntary work or caring for family competing with time for PA [10, 11], and an increased awareness of one's own aging, leading to fear of injury or the feeling of being too old for exercise [11].

However, most previous qualitative studies investigating PA during the retirement transition focused on PA barriers and facilitators in adults around retirement in general and not specifically on the changes in these barriers and facilitators during the retirement transition. For example, poor health has been identified as a barrier for PA in adults during the retirement transition [12–14]. Another example is a low personal value on recreational PA, which limits PA in retirement [11]. Nevertheless, these PA barriers did not specifically change during the transition from work to retirement. Consequently, while addressing these barriers is crucial for interventions in this age group, specifically addressing these barriers during the transition to retirement may not necessarily provide additional value.

A second limitation in earlier research is the absence of a specific connection between findings about changes in PA determinants during the retirement transition and a theoretical framework. By using such a theoretical framework, results would inform the development of future interventions more, as McDonald et al. [10] stated earlier. A valuable framework is the “behavior change wheel” (BCW), including the COM-B model [15] combined with the “theoretical domains framework” (TDF) [16, 17]. The COM-B model was chosen as the guiding theoretical framework for this study. The COM-B model is the most comprehensive and widely used behavioral change framework that combines and integrates the core elements of other behavioral change frameworks [15]. A major strength of the COM-B model is its ability to systematically identify the factors influencing the target behavior to highlight the most effective techniques for changing that target behavior and to design interventions. Within the COM-B model, which is an integral part of the BCW, the fundamental components are capability, opportunity, and motivation. These components interact with one another, influencing behavior, which, in turn, impacts these components once again. Capability is defined as “the individual's psychological and physical capacity to engage in the activity concerned.” Opportunity encompasses “all the factors that lie outside the individual that make the behavior possible or prompt it,” while motivation is described as “all those brain processes that energize and direct behavior, not just goals and conscious decision-making” [15]. Complementary to the COM-B model, the TDF serves as an integrative framework, defining 14 behavior change domains rooted in previously established psychological theories [16, 17]. Previous research has mapped these domains onto the COM-B model, providing a more detailed understanding of which behavior change domains may require modification to enhance the targeted behavior [17]. The COM-B and TDF have been used previously to investigate barriers and facilitators for several health behaviors in various target groups [18, 19].

Besides this, it is worth noting that many studies examining PA during the retirement transition were conducted over a decade ago [9–11]. Meanwhile, societal conditions and the birth cohort of new retirees have undergone significant changes. For example, the increased opportunities for remote work from home in various occupations, a trend that gained momentum during the COVID-19 pandemic, may have resulted in working adults spending more time at home, accompanied by a reduction in daily interactions with colleagues. This shift may potentially mitigate the impact of the transition from work to retirement on their daily activities, on daily PA, and on their social interactions.

The first objective of the present study is to investigate the perceived changes in PA behavior during the retirement transition in adults who retired in 2022 in Flanders, Belgium. The second objective is to explore their perceived changes in PA barriers and facilitators experienced and to link these with the BCW model and with possible intervention strategies. The local retirement context is an important issue in this research. At present, the legal retirement age in Belgium is 65. However, depending on the years worked, this can be earlier, starting from the age of 60. In fact, the effective labour market exit age in Belgium in the five-year period 2017–2022 was 61,1 for men and 61,3 for women. This is lower than the average exit age of 64,4 and 63,1 years across OECD countries for this period [20]. The disposable household income from retirees in Belgium exists for more than 80% out of public transfers, which is the highest rate of all OECD countries [20].

Understanding the specific barriers and facilitators of PA that undergo changes during the retirement transition is essential for designing PA interventions that effectively use the opportunities inherent to this life event.

## 2. Materials and Methods

The Standards for Reporting Qualitative Research Checklist (SRQR) [21] was used and is provided in Supplementary File 1.

### 2.1. Design

The present study used a qualitative, abductive design, since a combination of deductive and inductive methods was employed [22]. More specifically, the data collection and data analysis were partly deductively guided by the BCW [15] and the TDF [16, 17], while inductive coding and inductive theme development were also used. Face-to-face, individual, semistructured interviews were adopted as the data collection method to optimally capture the individual perceptions of changes in PA and determinants of changes in PA. The study was approved by the Social and Societal Ethics Committee of KU Leuven (G-2021-3176) and all participants provided written informed consent. The data were stored on a password-encrypted computer and documents including names or other sensitive data were encrypted with an extra password. The AVG/GDPR laws were followed as instructed by the ethics committee.

### 2.2. Recruitment and Sampling

The participants in the current study were selected from the sample of the Move into Retirement Study, an observational longitudinal quantitative study among a convenience sample of 76 participants. The objective of the Move into Retirement Study was to examine 24-hour behaviors (PA, sedentary behavior, and sleep) during the transition from work to retirement. Participants' 24-hour behaviors were measured with an accelerometer at four points: up to six months prior to retirement and at three, six, and twelve months after retirement. The interviews for the current study were conducted after the measurement of six months after retirement. Prior to the interview, no feedback was provided about any of the measures in the quantitative Move into Retirement Study.

Participants were recruited by advertisements on social media, through employers, through health assurances, by word of mouth, etc. The inclusion criterion for the Move into Retirement Study was going on retirement in 2022, while exclusion criteria were disability retirement, retirement because of health reasons, and athlete retirement. Participants had to plan a substantial decrease in working hours when they would retire but were allowed to continue some professional activity.

**Figure 1:**
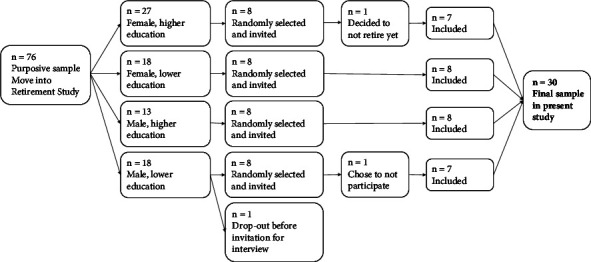
Flowchart of sample selection.

An overview of the sampling procedure is displayed in Figure 1. One participant from the larger Move into Retirement study dropped out of the study after the baseline measurement prior to retirement due to a lack of interest. After allocating the remaining 75 participants to four groups based on gender and educational level, eight participants were randomly selected out of each group, resulting in 32 participants who were invited to take part in the current study. This partly purposeful selection ensured that there was an equal distribution in gender and educational attainment to optimize heterogeneity in these characteristics and to obtain rich data. One participant chose not to take part in the interview study because he was not comfortable with talking about his experiences. Another person decided to continue working full-time just prior to retirement and was excluded from the study, resulting in a final sample size of 30 participants for the current study. Depending on data saturation after the data collection, more interviews could have been included with new participants.

### 2.3. Data Collection

Demographic and retirement-related factors used in this qualitative study were collected in the Move into Retirement study using questionnaires prior and after retirement and included gender, age, educational level (International Standard Classification of Education 2011) [23], occupational category (International Standard Classification of Occupations 2008) [24], subjective socioeconomic status (McArthur Scale of Subjective Social Status) [25], extent of living comfortably with one's income at preretirement and at twelve months postretirement, living situation (alone/with someone), partnership status, children, grandchildren, percentage of a full-time employment working prior to retirement, amount of hours/month paid and/or voluntary work after retirement, voluntariness of retirement, and perceived drop in financial income (“I had to adapt to a strong decrease in income”) (Table 1).

The interviews were conducted between September 2022 and July 2023, between 6 and 10 months after the retirement of the participant (median: 7.3 months). A time-use study by Sprod et al. found that changes in physical activity mainly occur from prior to retirement to three months after retirement, and are relatively stable from three months to twelve months after retirement [26]. At this point after retirement, it might be expected that the participants can still reflect sufficiently about changes from prior to after retirement, but retirement is not entirely new to them anymore, and there might have been some new habits embedded in their lives. The interviews took place at the university campus or at the participants' home, depending on the choice of the participant. In some cases, a masters' student was also present during the interview for educational purposes, always with prior consent of the participant. First, it was explained to the participants that we were interested in their experience and that they could not say anything wrong. They could take time to think whenever it was needed. The interview guide contained three open questions, each followed by general and specific prompts to obtain more detail and explanation. The integral interview guide is provided in Supplementary File 2. The open questions were as follows: “What changed in your life when you retired?,” “What changed in your PA when you retired?,” and “Do you think you have now more, less, or the same amount of PA?.” General prompts included questions such as “Why/how did this change for you?.” Specific prompts were based on the BCW [15] and TDF [16, 17] models. Example prompts were “Has something changed in the extent to which you plan beforehand to do PA?,” for example, when, where, what, and how much? for psychological capability in COM-B (BCW) and behavioral regulation in TDF. The interviews were pilot-tested with recently retired adults who did not participate in the study. The duration of the interviews ranged from 19 to 47 minutes with a mean duration of 30 minutes. The interviews were audio-recorded with the participants' consent by using a dictaphone and a smartphone as a backup. The recordings were transferred from the devices to a computer after the interview.

### 2.4. Data Analysis

The interviews were manually transcribed verbatim, pseudonymized, and coded inductively using the software NVivo (QSR International Pty Ltd, Version 13, 2020). The analysis was based on reflexive thematic analysis by Braun and Clarke using the traditional six steps: familiarizing with the data, generating initial codes, searching for themes, reviewing themes, defining and naming themes, and producing the report [27, 28]. After familiarization with the interview transcripts by transcribing and rereading, they were coded inductively line by line. Text passages representing coherent thoughts or ideas were assigned a code. Links between transcripts were made and clusters of codes were organized. Initial themes were created and reviewed several times. This was a cyclical process of going back and forth between the phases of the analysis until a theoretical saturation was reached. Regular meetings with all authors were held during the analysis process to discuss the coding and theme development. After this inductive phase, the identified themes were mapped onto the TDF [16, 17] and BCW models [15] by the whole author team together. A postpositivist tradition was applied throughout data collection and analysis [29]. In case of any disagreements within the team concerning coding or theme development, this was discussed with the research theme until a consensus was reached.

### 2.5. Research Team and Reflexivity

The first author of this publication conducted the interviews and coded and analysed the data. She is a 30-year-old female PhD candidate with a background in physiotherapy who followed training in qualitative research methods. She had already encountered most of the participants of the present study prior to the interviews because she collected data for the quantitative Move into Retirement Study. The other three authors of this publication are professors and guided the study process thoroughly. The research team members have different ages, ranging from 30 to 53, have different educational backgrounds (physiotherapy, epidemiology, psychology, and movement sciences), and have different genders, which reinforced rich discussion and different perspectives. Participants were informed in the informed consent form that we investigate PA because it is important for health and that the literature suggests that there might be changes in PA during the retirement transition.

### 2.6. Trustworthiness

Several measures were implemented to enhance the trustworthiness of the outcomes. The prior familiarization between (most) participants and the interviewer during the measurements for the quantitative study facilitated a comfortable atmosphere for open communication. We aimed to reduce bias due to the characteristics of the interviewer by self-reflection and self-awareness during the process. Analyst triangulation was used to increase the credibility and confirmability of the results. Information about participants, the context of the study, and sampling procedures is provided in this publication to improve the transferability of the results [30, 31].

## 3. Results

**Figure 2:**
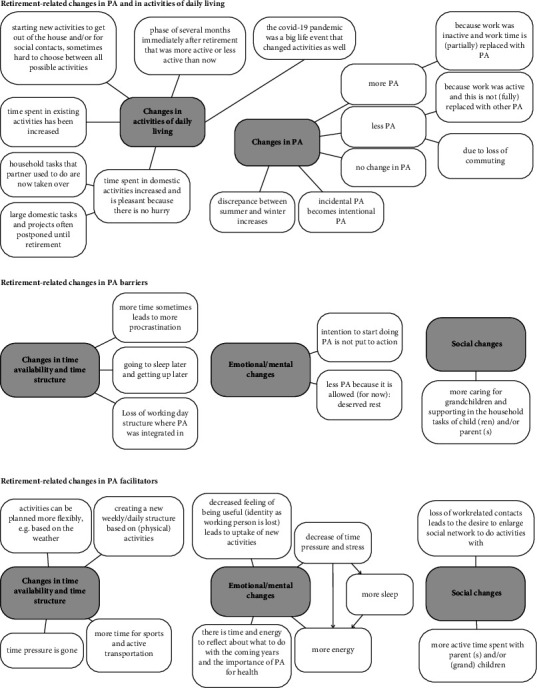
Visual overview (mind map) of the retirement-related changes in physical activity barriers and facilitators after inductive reflexive thematic analysis.

In total, 30 interviews were conducted (Figure 1). Data saturation was felt to be reached towards the final interviews. Based on the inductive reflexive thematic analysis, a mind map was developed with a schematic overview of the retirement-related changes in PA and its barriers and facilitators (Figure 2). The description of the results in this section is based on the structure of the mind map.

### 3.1. Retirement-Related Changes in PA and in Activities of Daily Living

Many participants perceived an increase in PA and this was mainly due to inactive work time being partially replaced with PA. However, other participants reported a decrease in PA and this was mainly due to active work time not being (fully) replaced with other PA, or due to the loss of active commuting time. A third group perceived no changes in their PA. Some participants mentioned that they had been doing PA more consciously since they retired.

Now, I do it by bike, even if it is still half an hour by bike. By car it would have been ten minutes, but uh, uh, those are things that I go about more consciously now, yes. (male, 66, higher education, six months since retirement)

Several participants mentioned that there was a phase of several months immediately after retirement that was more active or less active than at the time of the interview, which was six to ten months after the retirement transition.

As they say, so in the beginning you actually have a lot of activity, then you meet with this person and then with that one. (female, 63, higher education, seven months since retirement)

I do feel that I am now more rested than right after I, uh, stopped working. At that time, it was really, uh, like nothing really had to be done. While now you start to think, you are more into it, right? I think. (female, 66, lower education, nine months since retirement)

Next, multiple participants reported that compared to when they were not retired yet, their PA is higher in summer but lower in winter, or they expect this.

It (PA) will be less in winter, but in summer, I think it will double. I will be off more often and uh. Yeah, by bike, yeah, yeah, it will double. (male, 64, lower education, seven months after retirement)

It was almost summer then (when I retired), so at that time I was very active, from walking to cycling, I did everything I could. But in winter, that happens less. (female, 62, lower education, seven months after retirement)

Many participants started new activities, while others increased the time spent in existing activities. The reasons to start new activities were mostly to avoid being at home for the entire day, as this was perceived as unhealthy, and for social contact. Some participants found it hard to choose between all possible activities because of their interest in a broad range of activities.

I mainly think (that I do the voluntary work) to maintain social contact, like uh, I don't see myself sitting locked in my house, so to speak, from the morning until the evening, from Monday morning till Friday evening, and only cleaning house, uh, doing groceries and making dinner, and some chores. Not that it isn't fun to do so, I like to do those things, I always liked doing chores in and around the house, but no, no not exclusively. My world is, or, the world is not just eh, my house, you know. (male, 63, higher education, seven months since retirement)

Many participants mentioned having increased the time spent in domestic activities such as cleaning, tidying, doing repairs and “DIY” projects, and working in the garden. There were several reasons for this. Most participants enjoyed spending more time in these activities mostly because they could be performed at a leisurely pace. Also, larger projects around the house and garden were often postponed until after retirement, when there would be more time available for such projects. Finally, household tasks were often taken over from the life partner, especially if the partner was not yet retired.

I am gardening more now, it was not possible before, you know, because you returned home from work in the evening and you had to do some groceries or uh prepare dinner. As for household chores I now have the time for myself to take up some larger tasks. Uh, maybe tidy the whole basement and the attic. (female, 63, higher education, six months since retirement)

Some participants mentioned that the increased working from home during and after the COVID-19 pandemic has influenced their PA more than the retirement transition influenced their PA.

I worked from home a lot during the last few years, and I think it is an advantage for retirement, so the transition, my day was structured at home, and I am trying to stick to that structure. I have to say, before COVID-19, I went more often by bus. And during COVID-19, I started going on foot and that habit stayed with me. (female, 64, higher education, seven months since retirement)

That (dancing) was actually quite intense prior to COVID-19. And then COVID-19 hit and then we stopped and the only type of PA we still have now is walking when we are at the coast. And I thus started senior dancing in September. (female, 66, higher education, eight months since retirement)

### 3.2. Retirement-Related Changes in PA Barriers

Three main themes of retirement-related changes in PA barriers were identified and mapped across four of the COM-B components and four domains of the TDF (Table 2).

#### 3.2.1. Retirement-Related Changes in Perceived Time Availability and in Daily Time Structure

Retirement-related changes in perceived time availability and daily structure were important for participants who mentioned a decrease in PA after retirement. More specifically, the increase in time availability and the decrease in time pressure led to more procrastination for some participants (COM-B: psychological capability and TDF: behavioral regulation). Besides this, many participants mentioned going to sleep later and getting up later, which might lead to a decrease in PA as well (COM-B: physical opportunity and TDF: environmental context and resources).

My wife retired at the same time so we are searching for a way to divide our time so that it is a bit more normal, because now, we go to sleep at one am or half past one, so we have to look for a way to normalize that, but well eh, yeah.

(male, 64, higher education, seven months since retirement)

For some participants, the preretirement PA timing was linked to the working day structure and changes in that structure resulted in changes in PA (COM-B: physical opportunity and TDF: environmental context and resources).

When I was still working, I went for a walk at lunch three times a week. And now we still go for walks, but that pattern is gone. Like today, the weather is bad, you don't want to go for a walk and otherwise you were at your desk, and you went for a walk during the lunch break. (female, 63, lower education, six months since retirement)

#### 3.2.2. Retirement-Related Emotional/Mental Changes

Some participants who mentioned a decrease in PA expressed the intention to start doing PA but did not actually do it (yet) (COM-B: psychological capability and TDF: behavioral regulation).

Yeah, and now actual hobbies. I actually don't really know what hobbies I would have. No, I don't know. That will become clear in the future. Yeah, we still have not bought a bike, like I would like to do. For example, I would like to start doing yoga, which is something that is at the back of my mind. Maybe I can convince my husband to join a walking club. I don't want to do that alone so I will have to convince him. But at the moment, he is not really (laughs) willing to do that. (female, 63, lower education, six months since retirement)

Some participants reasoned that they deserved to rest for a while after all the working years and decreased their PA (COM-B: reflective motivation and TDF: intentions/goals).

I would not have wanted to be at home my whole life. So, it is not that I think, this is life, being retired, I think, that is life when you are 65 years old. Before, I loved to be active. But actually, after that long period I thought, it has been enough. Uh, it is, it is not expected of me anymore. And I don't expect it myself anymore either, actually. (female, 66, lower education, nine months since retirement)

#### 3.2.3. Retirement-Related Social Changes

Most participants mentioned caring more for grandchildren and/or parents and supporting the household tasks of the child(ren) and/or (a) parent(s) as well. However, this competed with time for (structured) PA in some participants (COM-B: social opportunity and TDF: social influences).

Usually, I go swimming once a week but this week it will not happen because I will have the grandchildren again. (female, 61, lower education, six months since retirement)

My daughter also lives further away so we go there often, we have a camper, so we go there with the camper often. And then you're sitting too, yeah, then we don't go for any walks or anything there, you know. Yeah, then we are visiting my daughter and then you cannot say, like, let's go for a walk, you know. (male, 62, lower education, seven months since retirement)

### 3.3. Retirement-Related Changes in PA Facilitators

Three main themes of retirement-related changes in PA facilitators were identified and mapped across four of the COM-B components and five domains of the TDF (Table 2).

#### 3.3.1. Retirement-Related Changes in Perceived Time Availability and in Daily Time Structure

Most participants who mentioned a PA increase after retirement mentioned enjoying the decrease in time pressure and the choice of daily structure. Logically, there was more time available for active transportation and for sports/exercise and to plan this more flexibly, e.g., based on weather conditions or on preferred timing (COM-B: physical opportunity and TDF: environmental context and resources).

Even when the weather is not really good, like now in winter, we go by bike and yeah then you wait for a day until the weather is good, you know. Because you think, like, we like doing so anyway and then you have some physical activity. Now you can say, like, if we cannot go on Saturday, then we'll go on Friday, right, it will be nice outside then. So that, uh, that is a great advantage. (female, 66, lower education, nine months since retirement)

However, some participants mentioned that the lack of daily structure induced stress and that they were attempting to find a new structure. This was sometimes achieved by planning PA (COM-B: psychological capability and TDF: behavioral regulation). Moreover, they related this to being willing to do “useful” activities and not being “lazy.”

I set my alarm at 7:30 AM, so I intend to, I have created some structure for myself, so that every day I know beforehand what I will do the next day. I have difficulty, uh, getting up in the morning and then deciding “so what am I going to do today,” it doesn't work like that for me. So, for example, I already plan some things in advance, like this room needs to be repainted. And if the weather is good, then I plan to do some things in the garden, or perhaps to go for a walk with some ex-colleagues. (female, 63, higher education, six months since retirement)

#### 3.3.2. Retirement-Related Emotional/Mental Changes

A feeling of freedom and a vacation feeling were very often mentioned by the participants who mentioned a retirement-related PA increase. The decreased time pressure and decreased work-related stress often induced better sleep and/or more energy availability, which can be used for PA (COM-B: physical opportunity and TDF: environmental context and resources).

I want to have more exercise because uhm, I actually already wanted that when I was working but I was just too tired, in fact. I got up very early every day, stressed by work, and I slept worse. And yeah, that is some kind of vicious circle, right, when you are too tired, yeah, then you don't feel like exercising anymore either. (female, 63, lower education, eight months since retirement)

Moreover, the increased available time and mental space, together with the new identity of “being retired” caused reflections on what to do with the coming years. Many participants realized that they wanted to stay or become active, mainly to stay healthy and independent (COM-B: psychological capability and TDF: memory, attention and decision process).

So when I see how my mother is deteriorating physically, well yeah, at 91 years old, not all of them are still in shape and uh, then I think like, it is important to work on that now to make sure that when you are older, you are not fully dependent on a wheelchair or something. Then now is the time, I think. (female, 64, higher education, seven months since retirement)

Lastly, a decreased feeling of being useful and the lost identity of being a working person sometimes led to the uptake of new activities to feel useful and to get a sense of purpose (COM-B: reflective motivation and TDF: social/professional role and identity).

I heard that from other people as well and I experience it myself. Or I need to be careful about that, so to speak, that you do not get the feeling that you are fruitless to society, so to speak, because you don't feel useful anymore. I think that is indeed an incentive to do some voluntary work, for example. (male, 63, higher education, seven months since retirement)

I don't feel comfortable with being all relaxed and reading the news on my phone and uh, yeah, that is not for me, I feel useless then, and then at noon I will feel like, really, what have I done today. (female, 63, higher education, six months since retirement)

#### 3.3.3. Retirement-Related Social Changes

Many participants who mentioned a PA increase did this as a way to enlarge their social network after retirement to compensate for their loss of work-related contacts (e.g., colleagues, customers, and patients). Some of the participants chose social activities that are physically active as well, such as walking or cycling with others, doing voluntary work, and doing sports (COM-B: social opportunity and TDF: social influences).

I feel that now, that I need more social contact compared to, before (retirement) I was exhausted when I got home and now it's like, I will more often talk to people, like, yeah, that would be nice, if you want to meet up, here is my phone number. (female, 62, lower education, doing paid work for approx. 27 h/month)

I first considered doing group sessions, to have some social contacts, but that is always in the evening. You can do many things (in the gym) individually that make you feel like you worked out, and sometimes, social contacts are made there because you are with many people in that room. (female, 63, higher education)

Apart from doing PA for socialising, participants who increased their PA levels after retirement also mentioned the opportunity to spend more active time with their (grand) children or with other family members (COM-B: social opportunity and TDF: social influences).

No, but like, joining a club or something, I don't feel the need for that and if I want to exercise, that is mostly with the children, with the grandchildren. It doesn't matter that they are there, that is not a problem. (female, 61, lower education, six months since retirement)

But now uhm, that I spend more time with them (the grandchildren), it is more varied, and we can also do a bicycle trip or in the neighbourhood. What I do then, actually, before they come here, I organise some kind of search game with little pieces of paper in the woods and then they have to go look for them. Things like that, those are nice. (female, 63, lower education, eight months since retirement)

## 4. Discussion

The two research aims of this study were to explore both the perceived changes in PA during the retirement transition as well as the changes in barriers and facilitators to engage in PA.

### 4.1. Perceived Changes in PA during the Retirement Transition

The majority of participants perceived an increase in overall PA after retirement, which is in line with previous qualitative findings [10], but in contrast with results from previous prospective quantitative studies about the effects of the retirement transition on overall PA [5, 6, 26]. These inconsistencies between qualitative and quantitative findings concerning the impact of retirement on total PA changes may be partly attributed to the difficulty of estimating changes in personal overall PA. More specifically, retirement coincides with an increase in time dedicated to more intentional, structured, and/or planned leisure time PA after retirement [5, 32, 33] and a decrease in commuting, active transportation, and occupational PA [5], which are more incidental and less conscious forms of PA. This means that the decrease in overall PA is likely attributable to a decrease in incidental, less conscious PA. Participants in the current study who perceived an increase in overall PA might have mainly increased their recreational PA and perceived this as an increase in total PA. This means that retirees should consider the changes in PA in all domains when reflecting on their overall levels of PA to avoid an overestimation of beneficial changes in PA. In future interventions, increasing understanding of PA levels through the use of self-monitoring tools and considering all domains and contexts of PA seems important, particularly during the retirement transition.

Concerning the changes in different domains of PA, two main findings emerged. First, the retirees in the current study reported that they allocate more time to domestic activities, such as household chores, gardening, and house repairs, which is consistent with previous quantitative findings [5]. Participants in the present study expressed enjoyment in engaging in these domestic activities and emphasized the freedom to do so without pressure or time constraints.

Second, our participants perceived an increase in active transportation after retirement, which is in line with several previous quantitative studies [34, 35], but in contrast with other studies [5, 26, 32]. While a few participants mentioned a loss in active commuting to work, the majority reported an increase in using active modes of transportation for tasks such as grocery shopping or visiting family and friends because there is more time available after retirement. This increase might be explained by the fact that (recreational) walking and cycling are among the most popular types of PA in older adults in Flanders (Belgium) [36]. Another explanation might be the increased use of electrical bikes [37, 38], especially in older adults, which are in Flanders especially popular among adults older than 55 [38].

Lastly, participants mentioned that the difference in their PA between summer and winter was larger since they retired, or they expected this, since most retirees were only retired for seven months. This was mainly due to the colder weather in winter and more dark times. Therefore, it might be important in PA interventions to pay extra attention to darker and colder periods.

### 4.2. Perceived Changes in PA Barriers and Facilitators

The second research aim of the present study was to explore the perceived changes in barriers and facilitators to engage in PA during the retirement transition. It is remarkable that despite the differences in study country and retirement contexts, most of the identified changes in barriers and facilitators were in line with previous studies by McDonald et al. [10] in the UK and Barnett et al. [11], a systematic review with studies from the UK, the USA, Germany, and Canada. The facilitators in agreement with those studies were an increase in time availability; increased energy levels; more flexibility to plan PA, for example during the daytime [10]; an increased need for social interaction; the need for a new routine; and an increased awareness of the importance of PA for one's own aging [11]. Barriers for engagement in PA which were confirmed in the current study encompassed increased procrastination [10], loss of daily structure [10, 11], and increased social responsibilities such as voluntary work or caring for the family which compete with time for PA [10, 11].

However, some previous findings were not reflected in the current study. First, the need for a new personal challenge was previously reported as a facilitator of PA during the retirement transition, mainly for men [11]. Some participants in our study attempted to find new purposeful activities but did not describe this as seeking a new challenge. Another barrier reported by McDonald et al. [10] was a decrease in financial resources and an increased awareness of the cost of PA. Some individuals in the present study expressed a certain degree of psychological stress regarding their financial situation but they did not associate this financial stress with their PA behavior. While the current study sample has a balanced representation of gender and educational levels, the financial situation of the participants in our sample might be better than the target population in Flanders. Moreover, the financial income of adults in Flanders is generally better compared to the average in Europe. In 2022, 8% of Flemish adults had an income below the poverty threshold, while the mean in the EU-SILC countries was 17%. Of the older adults in Flanders (65 and over), 13% had an income below the poverty threshold [39]. No participants in the current study had difficulties in living comfortably with the available income prior to retirement, while one participant did have difficulties after retirement. Only seven participants perceived a large decrease in income when they retired (Table 1). Third, fear of injury and the feeling of being too old for exercise were not mentioned by the participants in the current study, in contrast with Barnett et al. [11]. This might be due to the fact that the latter systematic review identified only studies focusing on recreational PA, rather than overall PA.

The present study established some additional determinants for PA engagement that changed during retirement. First of all, the flexibility to plan PA based on the weather conditions was a facilitating factor for participants who preferred outdoor activities. Given that, adverse weather conditions have been recognized as a significant barrier to adult PA [40], and the flexibility to plan activities after retirement at the most suitable time becomes important in overcoming this barrier, particularly in countries with fluctuating weather conditions. Furthermore, multiple participants mentioned that they spend more time with their family, in particular grandchildren, and that they were active together or that the care for the family was often physically active. Hence, the increase in time with family/grandchildren could be a facilitator for PA during the retirement transition. This is in line with Vermote et al. who reported that grandchild care could have a beneficial effect on levels of PA in older adults [41]. In contrast, in previous studies [10, 11] as well as the current study, participants mentioned also that caring for others competed with time for personal PA. Finally, many of the participants in the present study stated that they go to sleep later and get up later, which is a confirmation of previous findings regarding changes in sleep timing during the transition to retirement [42, 43]. This was a barrier for PA for some participants because exercise was previously planned in the morning, a time that is in retirement used for sleeping. However, better and more sleep has also been pointed out as a facilitator for PA in the present study because of higher energy levels during the day. Consequently, it might be advisable to prioritize sufficient sleep and to postpone morning PA if necessary.

### 4.3. Implications for Interventions

While the present study confirmed findings from previous studies investigating changes in barriers and facilitators during the retirement transition, it also highlighted new changes in barriers and facilitators to engage in PA. To add further to the literature, we linked the outcomes of the current study to BCW and TDF models and to the accompanying intervention functions described by Michie et al. [15]. While acknowledging the gap between the current study and intervention implementation, we discussed five potential intervention characteristics that could be important for interventions that make use of the opportunities of the transition from work to retirement. There is indeed a clear need for interventions to promote and maintain PA levels in the transition to retirement, since multiple quantitative observational studies reported a decrease in overall PA, while the systematic review by Baxter et al. identified only one PA intervention study which described the study sample as around the retirement transition [44]. Only a few more recent intervention studies have been reported [45–48], with varying definitions of “the transition to retirement” in terms of the time window around retirement.

First, several identified barriers and facilitators that changed during the transition to retirement were related to the COM-B category “psychological capability” (Table 2). Michie et al. describe that psychological capability can be achieved by focusing on education, training, and enablement and more specifically by imparting knowledge or understanding and training emotional, cognitive, and/or behavioral skills [15]. Therefore, an intervention should offer information to the target group that procrastination of PA is more common in retirement and could provide self-regulation techniques to overcome procrastination such as nudging, goal setting, and anticipating obstacles for PA. Also, cognitive restructuring techniques (e.g., positive reappraisal of the activity), tackling low self-efficacy, and increasing automaticity and routines for PA should be taken into account to overcome procrastination [49]. Previous research indeed showed that exercise procrastination (i.e., the voluntary delay of PA despite expecting to be worse off for the delay) [49, 50] is associated with lower overall PA [51]. Previous studies have shown that it is possible to increase PA in adults around the retirement transition by using goal setting and training self-regulation skills [45, 46]. In addition, interventions could stimulate reflection about aging and the importance of PA by using, for example, motivational interviewing techniques [52]. Moreover, the ability to create a new daily/weekly structure based on PA planning could be stimulated to compensate for the loss of time structure.

Second, many participants mentioned barriers and facilitators in the COM-B category “social opportunity.” They attempt to enlarge their social network to compensate for the (partial) loss of work-related contacts. Some join new organized sociophysical activities (e.g., walking or cycling clubs, fitness clubs, and physically active voluntary work) but others seek contact with old friends, neighbours, etc. to go walking or cycling together. The change in social life is in line with a Dutch study that found that people increase volunteering and organizational memberships when they retire [53]. An increased desire for social PA is beneficial for long-term PA engagement, since previous studies found that social support for PA is associated with higher PA engagement [54, 55]. Moreover, having membership in a social group, such as an exercise group, reduces the age-related declines in PA [56] and even premature death [57]. Therefore, framing future PA interventions in terms of both physical and social health benefits could be instrumental in encouraging retirees to incorporate more PA into their daily lives.

Third, in the COM-B category “social opportunity,” the increased care for and time spent with family, especially grandchildren, could be a barrier as well as a facilitator. Hence, it might be beneficial to stimulate PA for grandparents together with grandchildren by organizing intergenerational activities (e.g., activities in schools and sports clubs). In 2003-2004, 53.2% of Belgian grandparents were looking after grandchildren, which is higher than the mean of 43.6% in the countries in the SHARE study [58]. Moreover, for children born around 2015–2018 in Flanders, and who were regularly taken care of in formal or informal nonparental childcare, 18.3% of the childcare was provided by grandparents when the children were 0–3 years old [59]. Besides this, younger grandparents are more likely to take care of grandchildren and are often also experiencing the transition to retirement. Thus, the opportunity to use the time spent with grandchildren to be physically active in recent retirees is particularly interesting and should be explored further. Another facilitator identified in this COM-B category is an increased desire to expand the social network. Social PA for recent retirees should be enabled by organizing sufficient attractive activities for this age group and stimulating recent retirees to do PA together with others, in an organisation context or outside of an organisation context.

Fourth, “physical opportunity” also emerged as an important COM-B category. Having more available free time was a facilitator mentioned by many participants and indicates the opportunity for increasing PA inherent to the retirement transition. An advice to recent retirees could be to continue to reserve time for PA because there are indications that recreational PA increases shortly after retirement but decreases gradually afterwards [5]. PA interventions should also follow up on recent retirees for sufficient time. Not only the increased time for PA but also reduced stress levels, better sleep, and increased energy levels were beneficial for the enjoyment of PA. The opportunity for PA due to these higher energy levels should be highlighted towards retirees. As a barrier in this category, PA was sometimes linked to the working day structure (e.g., walking during lunch breaks or active commuting to work). The working day structure is not maintained in most retirees, so a new structure might help to maintain PA.

Fifth, related to the COM-B category “reflective motivation,” some participants mentioned a decreased feeling of being useful and a loss of the identity of being a working person. Most of the participants already counteracted this by taking up new purposeful activities such as voluntary work, exercise, and helping family members with household activities. Exercise was also mentioned as purposeful by participants because it is “not being lazy.” Some participants were still struggling with this feeling and did not take action (yet). In the BCW, reflective motivation can be achieved by education, persuasion (i.e., using communication to induce positive or negative feelings or stimulate action), and incentivisation [15]. In this light, it might be important to frame PA as a purposeful activity, either by implementing it in voluntary work, household tasks, and caring tasks or by framing PA as purposeful itself, because it is part of self-care. This seems a promising intervention function because another facilitator identified in the current and previous studies was an increased awareness of aging and the importance of PA for healthy aging [11]. Moreover, some participants have a lower intention to do PA because it is allowed in retirement to be less active, and it is a deserved rest after the working years. This might be tackled by education and persuasion.

Lastly, some participants mentioned that they used the first few months after retirement to rest and to not plan too much. It might be investigated whether it is more beneficial for long-term PA to let the recent retirees have this less active period or to try to convince them to create new habits immediately after retirement, or even prior to retirement.

### 4.4. Strengths and Limitations

The use of a theoretical framework and linking the findings to specific examples of intervention functions are particular strengths of the study, since there is a clear need for interventions in this target group [44]. Furthermore, the sample was heterogeneous regarding gender, educational and occupational background, and retirement circumstances, thereby increasing the richness of the obtained data. Lastly, the clear focus on the transition to retirement and the fixed timing of the interview shortly after retirement increased the chance of obtaining information specific to the transition.

However, we realize that the current study is not without limitations that need to be taken into account when interpreting the results. First, the specific characteristics of the research team may have biased data collection and analysis, as these are inherently subjective processes. Therefore, reflexive thematic analysis was used, where the researcher reflects on personal assumptions and how these might influence the results [28]. Second, participants might have been more conscious about their PA since they were participating in a quantitative study about PA from prior to their retirement and have been measured multiple times. Moreover, the six months after retirement accelerometer measurement took place shortly prior to the interview. This might have impacted their current experiences of PA. However, they did not receive any feedback about the quantitative measurements of their PA prior to the interview. Besides this, selection bias is likely to be present in the current study sample. The participants might be more than generally interested in PA and health as they were selected from a convenience sample. However, several recruitment channels were used to reach a population without a particular interest in PA (e.g., by recruiting via employers). To make the generalizability and the context of the participants clearer, the sample characteristics have been described extensively in Table 1. Lastly, the lack of cultural/racial diversity within the sample is a limitation that could be tackled in future studies.

## 5. Conclusion

The current study confirmed several changes in barriers and facilitators for PA engagement during the retirement transition, which increases the trust in these findings. Besides this, we identified additional factors that were not reported in previous studies. Future studies should build upon this knowledge and design PA interventions to overcome the new barriers for engaging in PA that manifest in early retirement and use the new facilitators that arise in early retirement. The transition to retirement might be a good opportunity to implement PA interventions, since daily life changes inherently to the transition.

## Figures and Tables

**Table 1:** Characteristics and context of the study sample.

	*n* (%)
*Preretirement*	
Gender	
Women	15 (50)
Men	15 (50)
Education [23]	
ISCED^1^ 0–4: low-intermediate education	15 (50)
ISCED 5–8: high education	15 (50)
Occupational group [24]	
ISCO^2^ 5–9 (manual workers)	9 (30)
ISCO 0–4 (nonmanual workers)	21 (70)
McArthur Scale Of Subjective Social Status, median [range] [25]	7 [5, 9]
Living comfortably with an income	
With a lot of difficulty	0 (0)
With difficulty	0 (0)
Reasonably	2 (7)
Good	19 (63)
Very good	9 (30)
Living status	
With someone	27 (90)
Alone	3 (10)
Partnership status	
Life partner	28 (93)
No life partner	2 (7)
Partner's working status	
Self-employed	13 (43)
Retired	13 (43)
Incapacitated/on sick leave	1 (3)
Seeking employment	1 (3)
Children	
No children	3 (10)
1 child	5 (17)
2 children	15 (50)
3 children	7 (23)
Grandchildren	
No grandchildren	11 (37)
1 grandchild	8 (27)
2 grandchildren	5 17)
>3 grandchildren	6 (20)
Percentage of full-time employment	
100	10 (33)
80%	8 (27)
40–80%	12 (40)

*Six months postretirement*	
Age, median (Q1, Q3)	63 (62, 65)
Reporting paid work, *n* (%)	5 (17) (15–100 hours/month)
Reporting voluntary work, *n* (%)	10 (33) (2–35 hours/month)
Voluntariness of retirement	
Voluntarily retired, *n* (%)	25 (83)
Pressured to go on retirement, *n* (%)	2 (7)
Partially voluntarily, partially pressured, *n* (%)	3 (10)
Perceived a large decrease in financial income	
Strongly disagree	5 (17)
Disagree	14 (48)
In between	3 (10)
Agree	6 (21)
Strongly agree	1 (3)

*Twelve months postretirement*	
Living comfortably with an income	
With a lot of difficulty	0 (0)
With difficulty	1 (3)
Reasonably	8 (27)
Good	13 (43)
Very good	8 (27)

^1^International Standard Classification of Education ^2^International Standard Classification of Occupations.

**Table 2:** Integration of the barriers and facilitators within the behavior change wheel framework [15] and theoretical domains framework [16, 17].

COM-B construct	Domain of theoretical domains framework	Retirement-related changes in PA barriers	Retirement-related changes in PA facilitators	BCW intervention functions [15]
Psychological capability	Knowledge			Education Training Enablement
	Memory, attention, decision processes		(i) There is time and energy to reflect on what to do with the coming years and the importance of PA for health	
	Behavioral regulation	(i) More time sometimes leads to more procrastination (ii) Intention to do PA is not put into action	(i) Counteracting the lack of daily/weekly structure by planning physical activities	

Physical capability	Skills			Training Enablement

Social opportunity	Social influences	(i) More time spent with grandchildren: competes for time for physical activity	(i) Attempt to enlarge social network to do activities with old and/or new contacts (ii) More active time spent with grandchildren	Environmental restructuring Enablement

Physical opportunity	Environmental context and resources	(i) PA was linked to the working day structure (ii) Going to sleep later and getting up later	(i) More time for sports and active transportation + flexibility to plan activities based on the weather conditions: possible increase in PA (ii) Having more free time leads to a decrease in time pressure and stress, better sleep, and more energy	Environmental restructuring Enablement

Reflective motivation	Beliefs about capabilities			Education Persuasion Incentiv isation
	Social/professional role and identity		(i) Decreased feeling of being useful (identity as a working person is lost) leads to uptake of new activities	
	Beliefs about consequences + optimism			
	Intentions + goals	(i) Lower intention to do PA because it is allowed for now (deserved rest) (sometimes only in the first few months postretirement)		

Automatic motivation	Emotion			Persuasion Incentivisation Coercion Environmental restructuring Modeling Enablement
	Reinforcement			

## Data Availability

The data used to support the findings of this study are available from the corresponding author upon reasonable request.

## Supplementary Materials

Supplementary Materials Supplementary file 1: Standards for Reporting Qualitative Research (SRQR) checklist. Supplementary file 2: interview protocol.
