# Supplementary material for: Exploring Changes in Barriers and Facilitators for Physical Activity during the Retirement Transition: A Qualitative Interview Study Based on the Behavior Change Wheel
Source: J Aging Res. 2024 Sep 16;2024:3257287. doi: 10.1155/2024/3257287 (PMC11419837; doi:10.1155/2024/3257287)
Supplement: Supplementary Materials — Supplementary file 1: Standards for Reporting Qualitative Research (SRQR) checklist. Supplementary file 2: interview protocol. [file 3257287.f1.zip › SupplementaryFile2-interviewprotocol.pdf]

Interview protocol

Introduction

I am going to ask you some questions about how you experienced 'going on retirement', if that is okay for you. I am going to record our conversation with an audio recorder, so that I can listen to it again afterwards. I will write it out afterwards without your name in it, so that no one knows that this is about you. Next, I want to add that you cannot say anything wrong here. Also, take your time to think about my questions if you need that. Is that okay for you? Do you have any questions?

Standard questions

1. What has changed in your life when you retired?
2. What has changed in your physical activity when you retired? Physical activity entails all forms of moving, so not only sports but also in the household, in the garden, at work, going somewhere on foot or by bike, and so on.
3. Do you think that you have now more, less or the same amount of physical activity?
4. Are there certain things that planned to do in retirement that you did not do yet?

➔ *These questions were extensively discussed and prompts were used to receive more explanation, to ask for reasons for certain changes in behaviour, etc.*

Extra prompts based on COM-B and TDF (only used when not enough information was gathered based on the previous questions)

**COM-B construct**	**COM-B Micro-Construct**	**Domain of Theoretical Domains Framework**	**Eliciting prompts**
Capability	Psychological	Knowledge	**Is there an association between physical activity and health? Is it important to have physical movement?**
		Memory, Attention and Decision Processes	
		Behavioural Regulation	(self-monitoring)<br>**Did something change in the extent to which you monitor/check for yourself how much physical activity you have and whether you have enough physical activity?**
			(breaking habit)

			**Is being active a habit for you? Is it something that you do automatically, without thinking about it? Or do you have to remind yourself to be active? Has this changed in comparison to before your retirement?**
			(action planning)<br>**Did something change in the extent to which you plan beforehand to be active? For example when, where, what en how much activity you will do?**
	Physical	Skills	**Are there activities that you can not do physically, or that you can not do easily? Did this change in comparison to before your retirement?**
Opportunity	Social	Social influences	(social support)<br>**Do the people around you influence you and your physical activity now differently than before your retirement?**<br>Extra prompts:<br>- Do you do physical activity toghether with others?<br>- Does your family stimulate you to be active? For example, to take over tasks from you so that you have more time, or by being interested in it?
			(subjective norms)<br>**What do people around you think about how active you are? Do they look at it differently than before your retirement?**
	Physical	Environmental context and Resources	(barriers and facilitators in the environment + material resources)<br>**Did something change in your environment compared to before your retirement? Something that might have influenced your physical activity?**<br>(materiaal aangekocht, nieuwe sportfaciliteit beginnen gebruiken, verhuisd, faciliteiten van het werk die weggevallen zijn, …)<br>(for example: purchase of new materials, started using new sports facility, moved house, facilities of the job that are not available anymore, etc)
Motivation	Reflective	Beliefs about Capabilities + Social/Professional Role & Identity	(identity)<br>**Did something change in the extent to which you see yourself as an active person?**<br>(self-efficacy)<br>**Did something change in how you handle it when your physical activity is challenged, for example because you are tired, because there is little time, because you have worries, etc?**<br>(perceived behavioural control: no prompt)
		Beliefs about Consequences	(See psychological – knowledge)

		+ Optimism	
		Intentions<br>+ Goals	**Do you expect that you will start being active in the future? Do you expect that you will start new physical activities?**
	Automatic	Emotion	(See self-efficacy - beliefs about capablities)
		Reinforcement	/
